# Plant invasions differentially affected by diversity and dominant species in native‐ and exotic‐dominated grasslands

**DOI:** 10.1002/ece3.1830

**Published:** 2015-11-17

**Authors:** Xia Xu, H. Wayne Polley, Kirsten Hofmockel, Pedram P. Daneshgar, Brian J. Wilsey

**Affiliations:** ^1^ Department of Ecology, Evolution and Organismal BiologyIowa State UniversityAmesIowa50011; ^2^ Grassland, Soil and Water Research LaboratoryUSDA‐ARSTempleTexas76502; ^3^ Department of BiologyMonmouth University400 Dedar AvenueWest Long BranchNew Jersey07764

**Keywords:** Climate change, dominant species, irrigation, precipitation pattern, native and exotic grasslands, phenology, plant invasions, species richness

## Abstract

Plant invasions are an increasingly serious global concern, especially as the climate changes. Here, we explored how plant invasions differed between native‐ and novel exotic‐dominated grasslands with experimental addition of summer precipitation in Texas in 2009. Exotic species greened up earlier than natives by an average of 18 days. This was associated with a lower invasion rate early in the growing season compared to native communities. However, invasion rate did not differ significantly between native and exotic communities across all sampling times. The predictors of invasion rate differed between native and exotic communities, with invasion being negatively influenced by species richness in natives and by dominant species in exotics. Interestingly, plant invasions matched the bimodal pattern of precipitation in Temple, Texas, and did not respond to the pulse of precipitation during the summer. Our results suggest that we will need to take different approaches in understanding of invasion between native and exotic grasslands. Moreover, with anticipated increasing variability in precipitation under global climate change, plant invasions may be constrained in their response if the precipitation pulses fall outside the normal growing period of invaders.

## Introduction

The invasion of habitats by plants is an increasingly serious global concern, causing modification and degradation of grasslands, especially under current climate change. Much attention has been paid to the invasibility in grasslands as affected by phenology and diversity (e.g., Naeem et al. 2000; Kennedy et al. 2002; Prevey et al. 2010; Concilio and Loik 2013). However, studies that compare plant invasions in native vs. exotic‐dominated grassland ecosystems are few (Kulmatiski 2006). Additionally, increasing variability in precipitation, as one important aspects of global climate change, is expected to alter the spread of invasive plants but in largely unknown ways.

The importance of phenology in shaping plant invasions has long been recognized. Phenological differences between resident species and invaders may contribute to invaders' success (e.g., Elton 1958; Losure et al. 2007; Wolkovich and Cleland 2011). Invaders tend to green up and bloom earlier and/or later in comparison with resident plant species (Wolkovich and Cleland 2011). The success of plant invasions may depend on efficient use of resources (e.g., light, space, and soil nutrients) that are temporally not used by resident plants (Davis et al. 2000). Successful establishment of exotic species may form exotic‐dominated novel system (Hobbs et al. 2006). Compared with native grasslands, exotic grasslands greened up much earlier in spring (Wilsey et al. 2011). This phenological difference may facilitate invasion in native communities due to the high availability of unused resources early in the year. In addition to phenology, species diversity is usually hypothesized to stabilize ecosystem function, implying that invasion will be lower in plant communities of high than low diversity (Elton 1958). Exotic‐dominated grasslands are often lower in diversity than native‐dominated grasslands (Wilsey et al. 2011; Martin et al. 2014), which sets up the prediction that exotics will be more invadable than native sites.

Experimental (e.g., Naeem et al. 2000; Kennedy et al. 2002; Prevey et al. 2010; Concilio and Loik 2013) and modeling (e.g., Case 1990) studies generally support that, in natural areas, species‐poor communities are more vulnerable to plant invaders than species‐rich communities (Elton 1958). For example, a positive relationship between diversity and stability is generally supported by empirical evidence (e.g., Tilman et al. 2006; Isbell et al. 2009; Cardinale et al. 2013). A possible explanation for these results is that asynchronous growth among species in species‐rich communities leads to greater resource use efficiency, and fewer resources are thus available to plant invaders. However, no relationship or negative relationships between the two variables were also found (Sankaran and McNaughton 1999; Polley et al. 2007; Grman et al. 2010; Sasaki and Lauenroth 2011). An alternative view to the diversity–stability hypothesis is that dominant species, rather than species diversity, influence ecosystem stability because strongly dominant species can be highly stable (Polley et al. 2007; Sasaki and Lauenroth 2011; Wilsey et al. 2014). In this case, dominant species could be more important than diversity, having vital impact on essential ecosystem functions such as ecosystem stability and primary productivity. For example, Wilsey et al. (2014) showed biomass CV was similar in species‐rich native and species‐poor exotic communities because dominant exotic species were more stable than dominant native species.

The seasonality and temporal variability of precipitation are projected to change in the Great Plains of the USA, accompanied by wetter summers in the southern Great Plains (Weltzin et al. 2003; Allan and Soden 2008; Seager and Vecchi 2010). In support of the wetter summer prediction, precipitation increased ca. 10% over the 20th century in the southern tallgrass prairie region (Karl and Knight 1998). Shifting precipitation to seasons when resident plant species grow relatively little may benefit plant invasions as competitive abilities of resident species are low. Invasion occurs when invaders can capture resources such as water that is temporally not used by resident plants. Therefore, precipitation changes in specific seasons could have serious consequences for invasion. Modeling studies predict that altered precipitation patterns will facilitate plant invasions (e.g., Kriticos et al. 2003; Pattison and Mack 2008; Prevey and Seastedt 2014). However, we know little about the importance of long‐term precipitation pattern of a region vs. short‐term rain pulses (e.g., wetter summer) as drivers in plant invasions.

In this study, we assessed how the invasion of plants into experimental grassland communities differing in grassland origin (all native vs. all exotic species) and irrigation (with or without summer irrigation) in Texas, USA. Grassland ecosystems, accounting for ca. 54% of the conterminous US, play a vital role in ecology and economics. They are ideal for addressing plant invasions with climate change as grasslands respond rapidly to global changes. Specifically, we ask: (1) do species invade native communities more because of their later green up compared to exotic communities? (2) do plant invasions match the long‐term local precipitation pattern or respond to short‐term rain pulses (irrigation)? and (3) do diversity and dominant species regulate plant invasions similarly in native vs. exotic communities? Our approach compares among multiple drivers predicted to impact invasion: native vs. exotic species composition, long‐term precipitation patterns vs. short‐term rain pulses, and diversity vs. dominant species.

## Materials and Methods

### Maintenance of exotic vs. native diversity experiment

This work was conducted at the USDA‐ARS Grassland, Soil, and Water Research Laboratory in central Texas. The site receives 878 mm of precipitation per year in a bimodal pattern with a large peak in the spring and a smaller peak in the autumn (Fig. S1). Soils are Vertisol usterts. A common garden experiment was established in 2007–2008 using 36 widely distributed native and exotic grassland species (Table S1). A two‐way factorial treatment arrangement (origin × irrigation) was applied to plots with a randomized block design, using random draws to vary species composition. We planted equal‐mass transplants (Wilsey et al. 2011) at a common density (72 individuals m^−2^) of either all native or all exotic species. Plant locations for all species were randomized separately in each plot. Native and exotic species were planted in monocultures and 9‐species mixtures that were populated using a paired species approach that controlled for phylogeny and growth form between pairs of native and exotic species. We used a large pool of native and exotic species, all exotic species used are already present in the Texas flora (Wilsey et al. 2011). This experiment allows us to effectively compare plant invasions into native and exotic communities while keeping soil type, disturbance rate, and phylogeny controlled.

Replication of mixtures within blocks was at both the species draw (composition) and true replicate levels. Draws were created by randomly sampling nine species from a pool of 18 native or 18 exotic species while keeping functional group relative abundance constant, with selection of a native species always being matched with its exotic pair. Four draws were included within each of the two blocks, and two replicates of each draw and treatment level were included for a total of 32 mixtures per block (4 draws × 2 origin × 2 irrigation × 2 replicates). Based on previous work on functional group proportions in nearby intact systems (Wilsey and Polley 2003; Polley et al. 2005), we planted 10 plants of each of four C_4_ grass species (each grass species equally abundant), eight plants of one C_3_ grass species, and six plants of each of four C_3_ forb species (one legume, three nonlegumes, all equally abundant) in each plot. Transplants established well during the establishment phase, with close to 100% survival observed by May 2008 (Wilsey et al. 2011). Monocultures of all 36 species were also used (2 blocks × 2 irrigation levels × 36 = 144) in the study. Irrigated plots were hand watered from mid‐July to mid‐August at a rate of 128 mm per month in eight increments of 16 mm beginning in 2008 (Fig. S1). Preliminary testing of our technique showed no lateral water flow across the flat field. Soil cores (5 cm in depth) from irrigated plots (mean water content of 21% in 2008 and 23% in 2009) had consistently higher soil moisture than those from control plots (mean water content of 7% in 2008 and 12% in 2009). Sampling to depth revealed that water content differed to at least 45 cm deep. Irrigation was during the summer to match predictions from global change models that predict increased rainfall in summer months (Allan and Soden 2008). For more detailed description of the MEND irrigation experiment, see Wilsey et al. (2011).

### Data collection

In 2009, we quantified plant invasion rate by sampling all invading plants. Here, we define plant invasions as plants that recruit into established communities and invasion as the successful establishment of invading species as measured by biomass accumulation, rather seed production or a second generation of nonresident species. In our plots, invaders mainly consisted of exotic species (*Lolium perenne, Bromus japonicus, Medicago lupulina,* and *Oxalis* sp.) and cosmopolitan weedy native species (*Ambrosia artemisiifolia, Plantago* patagonica*, Euphorbia* spp., and *Amphiachyris dracunculoides*). We sampled all invaders because it provides a more realistic estimate of invasion to sample the natural recruitment of all plants than to sample one or a few planted species (Wilsey and Polley 2002). Shoots and most of the roots of the species that invaded the native and the exotic plots were hand removed six times during the year (ca. days 50, 100, 130, 180, 230, and 330). Thus, these dates correspond to sampling intervals day 0–50, 50–100, 100–130, 130–180, 180–230, and 230–330. Removed invaders were transported to the laboratory, dried at 70°C, and weighed as invader biomass. Additionally, canopy cover of the plant communities was estimated on all plots on day 7, 51, 86, 120, and 169 in 2009. The date at which the canopy was estimated to have reached 50% cover was used to indicate the green up of vegetation (Fisher and Mustard 2007). Species richness was determined twice in each plot when estimating peak biomass (late June and October) using point intercept techniques (Wilsey et al. 2011). In this study, species richness was used as a measure of diversity at the plot scale. Long‐term (1914–2013) precipitation datasets were downloaded from the weather station at the Grassland, Soil, and Water Research Laboratory webpage (http://www.ars.usda.gov).

### Statistical analysis

Experimental response variables were analyzed using a mixed model ANOVA with PROC MIXED in SAS 9.2 (SAS Institute, Cary, NC) with origin (native vs. exotic) and irrigation as fixed factors, and block, draw (block) and its interactions as random factors (Wilsey et al. 2011). To compare plant invasions between monocultures and mixtures, we used log response ratios of invading plant biomass of each mixture and of component species in monoculture:

$$\ln\left(\frac{\text{mixture}}{\text{monoculture}}\right)=\ln\left(\frac{B_{\mathrm{mix}}}{\sum_{i=1}^{9}p_{i}\times B_{i}}\right)$$

where *B*_mix_ is invading plant biomass in mixtures, *p*_i_ is a percentage biomass of the *i*th species in the same mixture, and *B*_i_ is invading plant biomass of monocultures planted with the *i*th species in monocultures under exactly the same treatments. With this measure, negative values denote more plant invasions in monocultures, and positive values denote more plant invasions in mixtures. In the few instances where the invading plant biomass was 0 in the monoculture, calculations were based on the other species in the mixtures (Spehn et al. 2005). Responses of invading plant biomass and of the log biomass response ratio between mixtures and monocultures to treatments were analyzed with repeated‐measures ANOVA using a first‐order autoregressive AR(1) covariance structure, and Kenwood–Rogers corrections. The grand mean was compared to zero with a *t*‐test for an overall test of monoculture versus mixture differences. Green‐up dates based on the 50% canopy cover of plant communities and the related log response ratio were compared among treatments using the same model with one‐way ANOVA. To compare the importance of species richness vs. dominant species in regulating plant invasions between native and exotic communities, PROC REG with the statement of selection = AIC was performed. We tested a model with species richness as the only variable, then another with species richness and dominant species, then another with just dominant species, all compared simultaneously and the best model selected based on the lowest AIC value. Relationship of plant invasions with long‐term precipitation (1914–2013) was analyzed with PROC REG in SAS 9.2.

## Results

Phenology differed between native and exotic plots. Exotic mixtures on average greened up 18 days earlier than native mixtures (origin *F*_1, 21_ = 33.18, *P *<* *0.01, Table 1 and Fig. S2a). Exotic monocultures greened up 28 days earlier than native monocultures (origin *F*_1, 35.8_ = 17.76, *P *<* *0.01). This trend was found in all draws for mixtures (Fig. S3a) and in most species comparisons for monocultures (Fig. S3b) between natives and exotics. Interaction between origin and irrigation had little effect on green‐up dates (*P *>* *0.05). Monocultures greened up earlier than the mixtures across the treatments (mean ln ratio = 1.56, *P *<* *0.01, Fig. S2b).

**Table 1:** Results of mixed model ANOVA (*F*‐tests) that compared invading plant biomass (g m^−2^ day^−1^), green‐up dates, and the ln ratio of mixtures over monocultures of the two variables between native and exotic plots (Origin) that received summer irrigation or not (Irrig)

Source	Plant invasions						Green‐up date			
	Biomass		Early biomass		ln (mix/mono)		Green up		ln (mix/mono)	
	df	*F*	df	*F*	df	*F*	df	*F*	df	*F*
*Block*										
Origin	1, 11.3	2.03	1, 49	8.67**	1, 11.3	0.08	1, 21	33.18**	1, 13	1.12
Irrig	1, 13.1	3.25	1, 49	0.51	1, 9.47	0.01	1, 21	0.01	1, 46	0.23
O × I	1, 146	1.70	1, 49	1.78	1, 147	7.92**	1, 21	0.80	1, 46	1.28
Time	5, 192	13.78**	1, 49	34.62**	5, 147	13.37**				
Time × O	5, 147	1.64	1, 49	2.52	5, 147	1.94				
Time × I	5, 162	0.50	1, 49	0.10	5, 147	0.27				
Time × O × I	5, 147	0.39	1, 49	0.06	5, 140	0.72				

**: <0.01.

**Figure 1:**
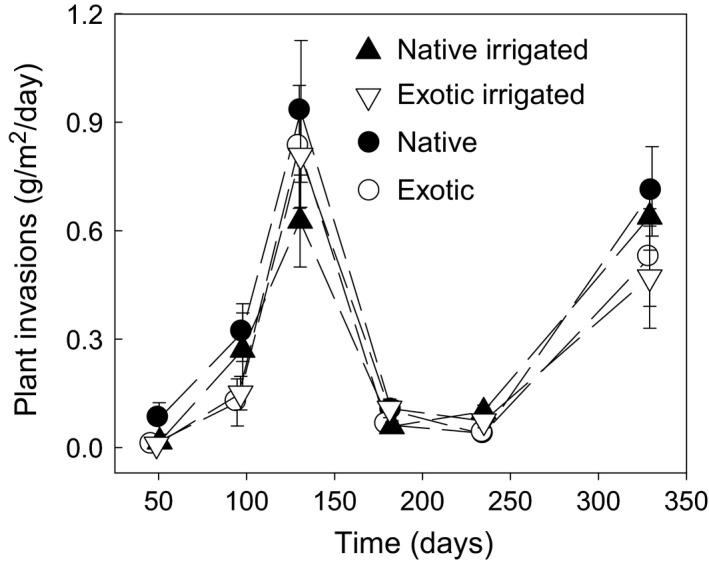
Dynamics of plant invasions across the six sampling times under the four treatments. Plant invasion rate (g m^−2^ day^−1^) was calculated based on invading plant biomass of each sampling time. Native and exotic refer to neighbors.

**Figure 2:**
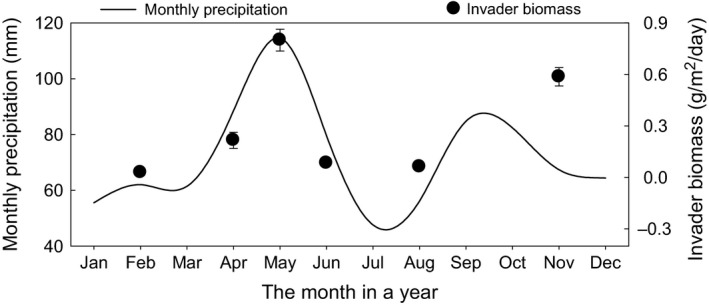
Adaptation of the plant invasion rate (across treatments) to the bimodal pattern of precipitation in Temple, TX. Monthly precipitation is calculated as the mean of 1914–2013 of each month with January beginning on January 1st.

**Figure 3:**
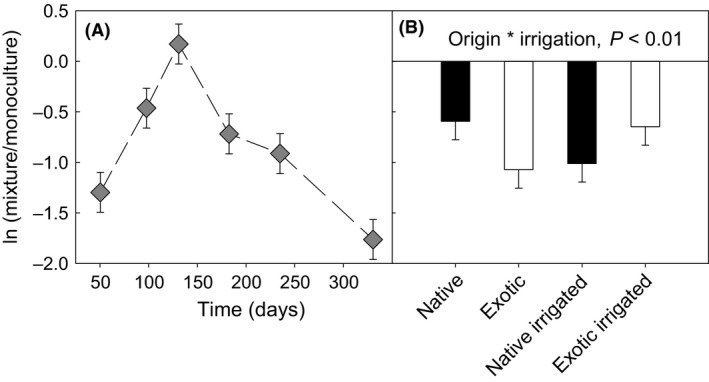
Dynamics of the ln ratio of mixture/monoculture on invading plant biomass across the six sampling times (A) and under different treatments (B). A negative ratio means that invading plant biomass was greater in monocultures than mixtures.

Plant invasions in native and exotic communities varied greatly across the sampling times in 2009 (time *F*_5, 192_ = 13.78, *P *<* *0.01, Table 1 and Fig. 1). Analyses based on the first two samplings showed that plant invasions were greater in native than exotic communities (origin *F*_1, 49_ = 8.67, *P *<* *0.01, Table 1 and Fig. 1). However, across the six sampling times, plant invasions did not differ between native and exotic communities either with or without summer irrigation (origin *F*_1, 11.3_ = 2.03, *P *>* *0.05, Table 1 and Fig. 1). Summer irrigation did not affect plant invasions across sampling times (irrig *F*_1, 13.1_ = 3.25, *P *>* *0.05, Table 1 and Fig. 1). Plant invasions showed two distinct peaks (intervals 100–130 and 230–330) that were consistent with the bimodal peaks in rainfall in the area (Fig. 2). Mean daily precipitation of 1914–2013 positively affected the invasion of plants (Fig. S4). As compared with mixtures, monocultures had significantly higher invasions according to the negative log response ratio of the invading plant biomass of mixtures over monocultures (mean = −1.55, SE = 0.40; *P *<* *0.01, Fig. 3A). The reduction in invasion in mixtures compared to monocultures was enhanced with irrigation in native communities, but was reduced in exotic communities (*F*_1, 147_ = 7.92, *P *<* *0.01, Table 1; Fig. 3B).

**Figure 4:**
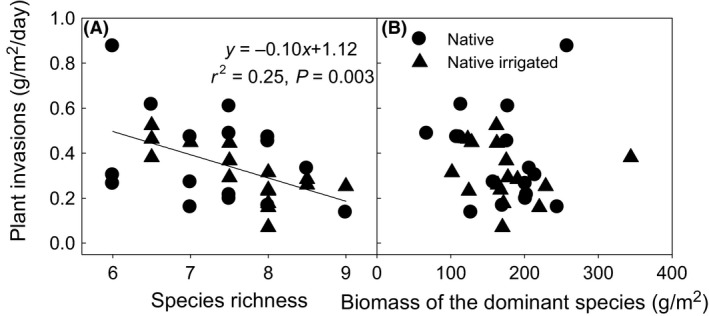
Plant invasions negatively affected by species richness of constituent species (A), but not by biomass of the dominant species (B) in native communities. Plant invasion rate (g m^−2^ day^−1^) was calculated based on the total invading plant biomass of the six sampling times.

**Figure 5:**
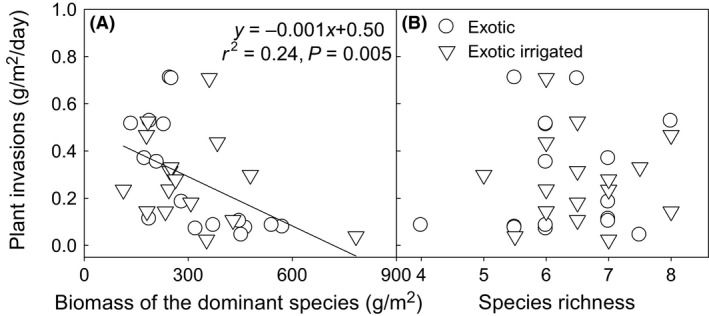
Plant invasions negatively affected by biomass of the dominant species (A), but not by species richness of constituent species (B) in native communities. Plant invasion rate (g m^−2^ day^−1^) was calculated based on the total invading plant biomass of the six sampling times.

**Figure 6:**
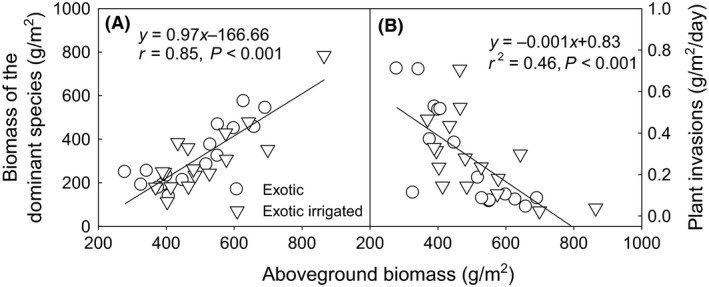
Relationships of aboveground biomass with biomass of the dominant species (A) and plant invasions (B) in exotic communities. Plant invasion rate (g m^−2^ day^−1^) was calculated based on the total invading plant biomass of the six sampling times.

The significant predictors of plant invasions were different between native and exotic communities. Plant invasions significantly increased with the increasing species richness in native mixtures (*P *<* *0.01, Fig. 4A), and biomass of the dominant species had little impact (Fig. 4B). In exotic communities, dominant species (*P *<* *0.01, Fig. 5A), rather than species richness (Fig. 5B), regulated plant invasions according to the negative relationship between biomass of the dominant species and the plant invasion rate. Plant invasions decreased as total biomass of exotic mixtures increased (Fig. 6).

## Discussion

We found that differences in phenology between invaders and established communities contributed to the success of invasion. Plant invasions were influenced by attributes of the communities that plants were invading. Consistent with the results of Losure et al. (2007), we found that plant invasions were not constant across time periods. Invasion rates followed the bimodal rainfall peaks of the local site (Temple, TX.), and invasion did not respond to our experimental water additions in the summer between peaks. This suggests that invaders might have adapted to the local precipitation regime and the mechanisms underlying this adaptation deserve further study. Perhaps most interestingly, the community level predictors behind invasion resistance differed between natives and exotic communities (species richness for natives vs. biomass of the dominant species for exotics). This suggests that we will need to take different approaches in understanding of invasion between native and exotic grasslands.

### Plant invasions differed between native and exotic grasslands

Early phenologies could enhance the success of exotic species (McEwan et al. 2009; Wilsey et al. 2011; Throop et al. 2012). Invasion of plants differed in native‐ and novel exotic‐dominated grassland ecosystems in early growing season partly because of phenological differences between native and exotic species. Plant invasions were smaller early in the season in exotic than native communities because exotic communities greened up and surpassed 50% canopy cover by an average of 18 days earlier than did native communities. Exotics may green up earlier than natives because successful exotic species have exploited the early spring growth period when native species are still dormant (Seabloom et al. 2003; DeFalco et al. 2007; Wilsey et al. 2011). Phenological differences between native and exotic communities contributed to higher plant invasions in native communities early in the growing season. A greater focus on how phenology may shape invasion and affect ecosystem functions is especially important with the advent of human‐induced climate change.

Plants preferentially invaded native communities early in the growing season; however, invasion did not statistically differ between community types across the full season. This suggests that there was a temporal shift in invasion, rather than an overall reduction in invasion rate in exotics. These results might be associated with species diversity, a major determinant of ecosystem invasibility (Hooper et al. 2005; Tilman et al. 2014). Higher species diversity in natives (Fig. S5) can increase resource uptake in ecosystems via a positive complementarity effect, which results from niche partitioning (Hooper et al. 2005; Isbell and Wilsey 2011). Previous studies in this and other grasslands have found that the complementarity effect was greater and temporal niche overlap was lower in these native than exotic communities (Isbell and Wilsey 2011; Martin et al. 2014). Greater plant invasions of native communities in the early part of the growing season thus may be countered by decreased invader growth resulting from greater resource depletion by natives in the later part of the growing season. We found that plant invasions were greater in monocultures than mixtures, further evidence of a diversity effect on invasion.

### Impacts of irrigation and precipitation pattern on plant invasions

Changes in precipitation during specific seasons were found to be particularly important in influencing plant invasion in some systems (Miller et al. 2006; Prevey and Seastedt 2014), but this was not supported in our study. In accordance with our results, summer irrigation was reported to have minor effects on invasion in a North America mixed‐grass Prairie (Blumenthal et al. 2008). This may be due to other factors, such as high temperatures in summer in Temple, TX, impeding invaders' growth. Additionally, irrigation did not significantly alter green up that could affect plant invasions, agreeing with the lack of a significant irrigation effect on plant invasions.

Modeling studies suggest that the invasion of non‐native species matches the local climate, although empirical evidence is limited (Kriticos et al. 2003; Pattison and Mack 2008). We found that the invaders in our system seemed to be adapted to the bimodal pattern of long‐term precipitation in Temple, TX. Plant invasions showed two peaks, closely tracking the precipitation peaks in spring and autumn. Importantly, this bimodal pattern could not be altered with water additions between the two peaks in the summer. In Iowa, where the historic precipitation pattern is unimodal, a single peak in invasion was found during late spring (Losure et al. 2007). Invaders may face strong abiotic selection pressures, and climate matching may be a prerequisite for a successful invasion (Moran and Alexander 2014). While precipitation is anticipated to vary greatly under global climate change with increasing intra‐ and interannual variability (Field et al. 2007; Li et al. 2013), plant invasions may be constrained in their response if the precipitation pulses are outside the normal peaks in growth.

### Diversity and dominant species differentially affected invasion

We found that resistance to plant invasions positively correlated with species richness in native communities, which is consistent with previous studies (e.g., Naeem et al. 2000; Kennedy et al. 2002). For example, resistance to *Bromus tectorum* invasion was found to be associated with higher species diversity in sagebrush steppe ecosystems in California and Idaho (Prevey et al. 2010; Concilio and Loik 2013). What was surprising was that the negative relationship between species richness and plant invasions did not hold true in exotic‐dominated systems, suggesting that diversity–stability mechanisms are altered (Wilsey et al. 2014). The counterview to the positive diversity–stability mechanism is that dominant species regulate ecosystem stability (Sasaki and Lauenroth 2011). Exotic mixtures with low species richness did not necessarily have low resistance to plant invasions. Rather, biomass of the dominant exotic species negatively influenced plant invasions partly by increasing community biomass. This suggests that the processes of invasion into native communities may not be directly applicable to exotic communities.

## Conflict of Interest

None declared.

## Supporting information


**Table S1.** List of species used in the experiment.
**Figure S1.** Precipitation patterns of the long‐term (1914–2013) and the year 2009 at the Temple experimental site.
**Figure S2.** Green‐up date in mixtures (a) and the ln ratio of mixture/monoculture on green‐up dates (b) across treatments. A ratio 0 in panel b means mixtures and monoculture greened up on the same day.
**Figure S3.** The difference in the green‐up dates for exotics compared to natives (exotic‐native) in mixtures (a) and monocultures (b).
**Figure S4.** Mean daily precipitation of 1914–2013 positively influenced weed invasion across sampling times. Weed invasion rate (g m^−2^ d^−1^) and mean daily precipitation (mm) were calculated based on each weed sampling period.
**Figure S5** Species richness (a), evenness (b), Simpson's diversity (c) and dominance (d) under different treatments in mixtures in 2009. Different letters indicate statistically significant differences at *P *<* *0.05.
