# Supplementary material for: Plant invasions differentially affected by diversity and dominant species in native‐ and exotic‐dominated grasslands
Source: Ecol Evol. 2015 Nov 17;5(23):5662–70. doi: 10.1002/ece3.1830 (PMC4813100; doi:10.1002/ece3.1830)
Supplement: Supplementary file 1 — Table S1. List of species used in the experiment. Figure S1. Precipitation patterns of the long‐term (1914–2013) and the year 2009 at the Temple experimental site. Figure S2. Green‐up date in mixtures (a) and the ln ratio of mixture/monoculture on green‐up dates (b) across treatments. A ratio 0 in panel b means mixtures and monoculture greened up on the same day. Figure S3. The difference in the green‐up dates for exotics compared to natives (exotic‐native) in mixtures (a) and monocultures (b). Figure S4. Mean daily precipitation of 1914–2013 positively influenced weed invasion across sampling times. Weed invasion rate (g m−2 d−1) and mean daily precipitation (mm) were calculated based on each weed sampling period. Figure S5 Species richness (a), evenness (b), Simpson's diversity (c) and dominance (d) under different treatments in mixtures in 2009. Different letters indicate statistically significant differences at P < 0.05. [file ECE3-5-5662-s001.doc]

**Supplementary materials**

**Weed invasion as differentially affected by phenology and stability in native- and exotic-dominated grasslands**

Xia Xu, H. Wayne Polley, Kirsten Hofmockel, Pedram P. Daneshgar, Brian J. Wilsey

**Supplementary Tables**

Table S1 List of species used in the experiment. Exotic and native species were paired based on phylogeny and growth form. Only four C_3_ grass species were used due to their paucity in the system.

| Native species | Family | Origin | Exotic species pair |
| --- | --- | --- | --- |
| *C_4_ grasses:* |  |  |  |
| *Schizachyrium scoparium^1,4,5^* | *Poacae* | *Asia* | *Bothriochloa ischaemum^1,2,6^* |
| *Buchloe dactyloides^2^* | *Poacae* | *Africa* | *Cynodon dactylon^2^* |
| *Sporobolus asper^1,2^* | *Poacae* | *Africa* | *Eragrostis curvula^6^* |
| *Panicum virgatum (short ecotype) ^4^* | *Poacae* | *Africa* | *Panicum coloratum^6^* |
| *Eriochloa sericea^4^* | *Poacae* | *South America* | *Paspalum dilatatum^2^* |
| *Sorghastrum nutans^1,2,4^* | *Poacae* | *Mediterranean* | *Sorghum halapense^1,2^* |
| *C_3_ grasses:* |  |  |  |
| *Nasella luecotricha^2^* | *Poacae* | *Europe* | *Dactylus glomerata^6^* |
| *Elymus canadensis^4,5^* | *Poacae* | *Europe* | *Festuca arundinacea^6^* |
| *C_3_ Forbs:* |  |  |  |
| *Ratibida columnifera^4,5^* | *Asteraceae* | *Eurasia* | *Leucanthemum vulgare^6^* |
| *Marshallia caespitosa^7^* | *Asteraceae* | *Europe* | *Taraxacum officinale^2^* |
| *Vernonia baldwinii^4,7^* | *Asteraceae* | *Eurasia* | *Cichorium intybus^6^* |
| *Salvia azurea^1,4^* | *Lamiaceae* | *Eurasia* | *Nepata cataria^6^* |
| *Ruellia humilis^6^* | *Acanthaceae* | *Mexico* | *Ruellia brittoniana^7^* |
| *Monarda fistulosa^4,7^* | *Lamiaceae* | *Eurasia* | *Marrubium vulgare^6,7^* |
| *C_3_ Leguminous forbs:* |  |  |  |
| *Dalea purpurea^3,4^* | *Fabaceae* | *Eurasia* | *Lotus corniculatus^6^* |
| *Dalea candidum^4^* | *Fabaceae* | *Europe* | *Trifolium repens^6^* |
| *Desmanthus illinoensis^4^* | *Fabaceae* | *Asia* | *Medicago sativa^6^* |
| *Astragalus canadensis^6^* | *Fabaceae* | *Mediterranean* | *Coronilla varia^6^* |

Propagule sources:

*^1^ Field collected seed ^2^ Field collected vegetative*

*^3^‘Wildseed Farms’ seed ^4^‘Native American Seed Co.’ seed*

*^5^ Field collected seed from ‘Sweet Briar Nursery’ ^6^ Other company seed*

*^7^ Other company, vegetative*

**Supplementary figure legends**

**Figure S1** Precipitation patterns of the long-term (1914-2013) and the year 2009 at the Temple experimental site.

**Figure S2** Green-up date in mixtures (a) and the *ln* ratio of mixture/monoculture on green-up dates (b) across treatments. A ratio 0 in panel b means mixtures and monoculture greened up on the same day.

**Figure S3** The difference in the green-up dates for exotics compared to natives (exotic-native) in mixtures (a) and monocultures (b).

**Figure S4** Mean daily precipitation of 1914-2013 positively influenced weed invasion across sampling times. Weed invasion rate (g m^-2^ d^-1^) and mean daily precipitation (mm) were calculated based on each weed sampling period.

**Figure S5** Species richness (a), evenness (b), Simpson’s diversity (c) and dominance (d) under different treatments in mixtures in 2009. Different letters indicate statistically significant differences at *P*<0.05.

Fig. S1

Fig. S2

Fig. S3

Fig. S4

Fig. S5
